# Influence of *Glycyrrhiza glabra* Extract on Growth, Gene Expression of Gut Integrity, and *Campylobacter jejuni* Colonization in Broiler Chickens

**DOI:** 10.3389/fvets.2020.612063

**Published:** 2020-12-22

**Authors:** Doaa Ibrahim, Alaa H. Sewid, Ahmed H. Arisha, Amir H. abd El-fattah, Adel M. Abdelaziz, Omar A. Al-Jabr, Asmaa T. Y. Kishawy

**Affiliations:** ^1^Department of Nutrition and Clinical Nutrition, Faculty of Veterinary Medicine, Zagazig University, Zagazig, Egypt; ^2^Department of Microbiology, Faculty of Veterinary Medicine, Zagazig University, Zagazig, Egypt; ^3^Department of Biomedical and Diagnostic Sciences, University of Tennessee, Knoxville, Knoxville, TN, United States; ^4^Department of Physiology, Faculty of Veterinary Medicine, Zaagazig University, Zagazig, Egypt; ^5^Department of Animal Physiology and Biochemistry, Faculty of Veterinary Medicine, Badr University in Cairo (BUC), Badr City, Egypt; ^6^Department of Animal Wealth Department, Faculty of Veterinary Medicine, Zagazig University, Zagazig, Egypt; ^7^Faculty of Veterinary Medicine, Veterinary Educational Hospital, Zagazig University, Zagazig, Egypt; ^8^Department of Microbiology, College of Veterinary Medicine, King Faisal University, Hofuf, Saudi Arabia

**Keywords:** liquorice extract, tight junction, performance, therapy, broiler

## Abstract

Phytogenic feed additives have been gaining considerable interest due to their ability to improve gut health and thereby performance of broiler chickens. The impact of *Glycyrrhiza glabra* (licorice) extract (GE) on expression of genes coding for tight junction proteins and gut protection and *Campylobacter jejuni* colonization in broilers has not been discussed until now. Thus, the current study assessed the effective dose of GE for maximum growth in broiler chickens, clear-cut molecular mechanisms related to integrity and health of intestine, and controlling *C. jejuni* colonization. Over a 35-day feeding period, a total of 500 Ross broiler chicks were allocated to five groups; the first group was fed a control diet without GE and the second group to the fifth group were fed a control diet with GE (0.25, 0.5, 1, and 2 g/kg of diet); each group comprised 100 chicks with 10 replicates (10 birds/replicate). Birds fed GE had an improved body weight gain and feed conversion ratio. Furthermore, the highest body weight gain was observed in the group that received 1 g/kg of GE (*P* < 0.05). The expression of genes coding for tight junction proteins [occludin and junctional adhesion molecules (JAM)] was upregulated in all groups supplemented with GE. Moreover, birds fed 1 g/kg of GE exhibited the maximum gene expression of occludin and JAM [0.2 and 0.3 fold change, respectively (*P* < 0.05)]. In relation to enterocyte protective genes [glucagon-like peptide (GLP-2) and fatty acid-binding protein (FABP-6)], use of GE significantly upregulated expression of GLP-2 gene with 0.8 fold change in 2 g/kg of the GE supplemented group (*P* < 0.05) while the expression of FABP-6 gene was not affected by GE supplementation (*P* > 0.05). After challenge with *C. jejuni*, the expression of mucin (MUC-2) gene was upregulated and the inflammatory markers such as Toll-like receptors (TLR-4) and interleukin (IL-1β) were downregulated with increasing level of supplemented GE (*P* < 0.05). The mean log^10^ count of *C. jejuni* in cecal samples after 7 days post-infection by culture and real-time qPCR was decreased in groups fed GE in a dose-dependent manner (*P* < 0.05). In addition, the highest reduction of *C. jejuni* count in cecal samples by culture and real-time qPCR was observed in the group fed 2 g/kg of GE (2.58 and 2.28 log^10^ CFU/g, respectively). Results from this study suggested that *G. glabra* extract (1 g/kg) improved growth performance of broiler chickens, as well as influenced the maintenance of intestinal integrity and reduced *C. jejuni* shedding from infected birds.

## Introduction

The gastrointestinal tract (GIT) not only plays a role in nutrient digestion and absorption but also acts as a metabolic and immunological organ. The intestinal epithelium forms a barrier that is essential for animal health. To keep and regulate the integrity of the epithelial cell barrier, the cells are connected by tight junction (TJ) complexes consisting of TJ proteins [occludin and junctional adhesion molecules (JAM-2)] (1, 2). Moreover, the enterocyte protection was controlled by expression of glucagon-like peptide (GLP-2) and Toll-like receptors (TLR-4), mucin-2 (MUC-2), and fatty acid-binding protein (FABP-6) genes (3). When the intestinal barrier integrity is interrupted, luminal substances can pass through the intact barrier and enter the body, causing an immune response such as inflammation, or impair animal health (4). Also, decreasing the intactness of intestinal integrity could result in increasing the bacterial adherence to the intestinal mucosa, translocation of bacteria, and susceptibility to infection from opportunistic bacteria and nutrient malabsorption (5). The GIT integrity can be influenced by dietary factors and intestinal pathogens (*Escherichia coli, Clostridium perfringens, Salmonella Typhimurium, Campylobacter*, etc.), causing alteration in TJ protein expression and disruption of enzymatic protein (6). *Campylobacter jejuni* is a gram-negative microaerophilic bacterium and is a well-known main causative agent for human acute gastrointestinal disorders (7). It was estimated that reduction of *C. jejuni* in chicken GIT by 2 log^10^ potentially decreased the frequency of human campylobacteriosis by 30-fold (8). Unfortunately, there are no effective control measures for *C. jejuni* in chickens. The poultry industry has made substantial efforts for decreasing in-feed antibiotics; thus, new strategies for controlling *C. jejuni* in poultry are required. On the other hand, certain dietary factors exert a protective effect on the intestinal barrier (9). The development of microbial resistance to antibiotics, the presence of antibiotic residues in animal products, and the impact of these residues on human health have encouraged the use of natural plant extract as alternative feed additives in the poultry industry in recent years (10). Additionally, phytogenic feed additives (PFA) and plant-derived agents have been gaining considerable interest lately due to their ability to improve poultry performance by enhancing nutrient utilization, sustaining a healthy gut integrity, and reducing pathogen load (11). PFA are beneficially modulating the intestinal microbiota recovery from intestinal challenge (12) due to their antimicrobial and antioxidant properties (13, 14). Also, dietary PFA can influence the maintenance of GI integrity via altering expression of genes coding TJ proteins and modulating the cellular immune system (15). Among these PFA is the *Glycyrrhiza glabra*, which is a traditional medicinal plant documented across the world for its biological and pharmaceutical properties. Its extract comprises several active compounds including more than 20 saponin triterpenes, 300 flavonoids, and additional components such as coumarins, sugars, starch, amino acids, choline, tannins, phytosterols, choline, and ascorbic acid. In addition, *G. glabra* extract (GE) has been registered to have immunomodulatory, antimicrobial, anti-inflammatory, antioxidative, and radical scavenging activities (16). Also, increasing gastric mucus secretion and antiulcer activity has been reported for GE (17). Dietary supplementation of GE and *Astragalus membranaceus* clearly upregulated the expression of growth-related genes as IGF-1 (18) in yellow perch. Moreover, GE extract contains bioactive chemicals such as glycyrrhizin, glycyrrhizinic acid, glabridin, glabrene, and glabrol, which had a potential antibacterial against many bacterial strains either gram positive or gram negative such as *B. cereus, E. coli, S*. *Typhimurium, Staphylococcus aureus, V. cholerae*, and *B*. *subtilis* (19). Also, GE shows highest antiadhesion activity against *C. jejuni* (20). The possible mechanism of GE on growth performance of broiler chickens could be attributed to the enhancement of GI integrity via altering expression TJ proteins, nutrient absorption, and intestinal immune system (15). *G. glabra* extract can play an important role in intestinal health owing to its active principal content; however, the mechanisms on how to regulate gene expression relating to intestinal integrity and *C. jejuni* colonization are still not understood in broilers yet. Thus, the aim of this study was to provide an evaluation for the efficacy of GE on growth performance; expression of occludin, JAM-2, FABP-6, GLP-2, TLR-4, and MUC-2 genes; and resistance against *C. jejuni* challenge in broiler chickens.

## Materials and Methods

The care and management of birds and experimental procedures were in compliance with ethics and guidelines of the Institutional Animal Care and Use Committee of the Faculty of Veterinary Medicine at Zagazig University.

### Experimental Birds, Diet, and Design

A total of 500 male broiler chicks (ROSS 308), 1 day old, were obtained from commercial hatchery with an average body weight of 45 ± 1 g. The experiment was performed in the broiler experimental unit at the Faculty of Veterinary Medicine at Zagazig University. Chicks were reared in the floor of 50 pens, weighed, and allocated randomly to five groups with 10 replicate pens consisting of 10 chicks per pen. *Glycyrrhiza* extract powder was added to the prepared basal diet and thoroughly mixed at concentrations of 0, 0.25, 0.5, 1, and 2 g/kg of diet for 35 days (control, GE0.25, GE0.5, GE1, and GE2, respectively), at the time feeding. Lighting regime was 24 h from days 1 to 3 and then 23 h lighting was applied up to the end of the experiment. The temperature was adjusted to 33 ± 1°C for the first 3 days, and then decreased by 3°C each week until it reached 24°C at the end of the experimental period and humidity was maintained around 60% throughout the whole experiment. The basal diet was formulated in mash form according to nutritional specifications of ROSS broilers (21). Diets were formulated as starter–grower (1–20 days) and finisher (21–35 days). All broiler chicks were given *ad libitum* access to water and feed. The feed ingredient and chemical composition of the control diet are listed in Table 1. Mortality was recorded daily throughout the study period. The proximate analysis of the feed ingredients was carried out according to the standard procedures of the Association of Official Agricultural Chemists (22). *G. glabra* extract was purchased from Shaanxi Sinuote Biotech Co., Ltd, China (product name: Glabridin 40% Licorice extract). The analysis of the product based on the manufacturing company is glabridin 40% by HPLC, glycyrrhizic acid/glycyrrhizin acid/glycyrrhizinate/glycyrrhizin 20–98% by HPLC and glycyrrhizic acid ammonium salt 98% by HPLC. Chemically, licorice extract is a triterpenoid saponin glycoside being either the Ca2+ or K+ salt of glycyrrhizic (or glycyrrhizinic) acid. Upon hydrolysis, the licorice root extract loses its sweet taste and is converted to the aglycone glycyrrhetinic acid plus two molecules of glucuronic acid. The acid form is not particularly water soluble, but its ammonium salt is soluble in water at pH >4.5.

**Table 1:** Ingredients and chemical composition of the basal diets (as DM).

**Ingredients %**	**Stater**	**Grower to finisher**
Wheat grain	46.7	53.2
Barely grain	15	15
Soybean meal	22.8	15.5
Corn gluten	6.8	7
Soybean oil	4.5	5.3
Calcium carbonate	1.48	1.28
Dicalcium phosphate	1.17	1.17
Common salt	0.3	0.3
Premix^*^	0.25	0.25
DL-Methionine, 98%	0.3	0.3
Lysine, HCl, 78%	0.5	0.5
Toxenil	0.1	0.1
Sodium bicarbonate	0.1	0.1
**Chemical composition**		
ME, kcal/kg	3,100	3,200
DM, %	89.96	90.03
OM, %	93.22	93.83
CP, %	22.65	20.05
EE, %	6.28	7.15
CF, %	3.19	3.12
Ca, %	0.96	0.87
Available *P*, %	0.45	0.45
Lysine, %	1.39	1.20
Methionine, %	0.67	0.64

* *Supplied, kg/diet: Vitamin A, 12,000 IU; Vitamin D3, 2200 IU; Vitamin E, 26 IU; Vitamin K3, 6.25 mg; Vitamin B1, 3.75 mg; Vitamin B2, 6.6 mg; Vitamin B6, 1.5 g; Pantothenic acid, 18.8 mg; Vitamin B12, 0.31 mg; Niacin, 30 mg; Folic acid, 1.25 mg; Biotin, 0.6 mg; Fe, 50 mg; Mn, 60 mg; Cu, 6 mg; I, 1 mg; Co, 1 mg; Se, 0.20 mg; Zn, 50 mg; Choline chloride, 500 mg*.

*DM, dry matter; OM, organic matter; ME, metabolic energy; EE, ether extract; CF, crude fiber; Ca, calcium; P, phosphorus*.

### Growth Parameters

All birds in each replicate were weighed at day 1, day 21, and day 35 of age. The feed intake per replicate was recorded by subtracting the remaining feed weight from the initial feed weight during starter and grower phases. Mortality and feed conversion ratio (FCR) [feed intake (g/bird)/weight gain (g/bird)] were calculated within starter, grower, and overall phases (days 1–35). Protein efficiency ratio [body weight (g/bird)/protein intake (g/bird)] was calculated 1–35 days of age.

### Sample Collection and Analytical Procedures

At the end of the experiment (day 35), 50 birds per group (5 birds per replicate pen) were randomly selected, weighed, euthanized, and slaughtered by cervical dislocation, and then carcasses were opened immediately.

For molecular analysis, small intestine (Jejunal part) was separated and digesta was squeezed out from it and rinsed three times in phosphate buffer saline (PBS), 1 cm from distal jejunum immediately prior to Meckel's diverticulum being dissected and kept in TRI Reagent at −80°C until real-time PCR analysis for genes coding TJ proteins and gut protection before challenge (*n* = 50 per group).

### Intestinal Real-Time PCR for Tight Protein Junction, Inflammatory, and Gut Protective Genes

Total RNA from intestinal samples was extracted by RNeasy Mini Kit (Qiagen, Cat. No. 74104). The quantity and purity of total RNA were measured using a NanoDrop ND-8000 spectrophotometer (Thermo Fisher Scientific, Waltham, United States) with the following cycling parameters: initial denaturation 95°C/15 min for 1 cycle, denaturation 95°C/15 s, annealing 60°C/60 s extension 72°C/60 s for 40 cycles, with data acquisition occurring at the 60°C step (23). Complementary DNA (cDNA) was obtained by reverse transcription of isolated RNA samples using RevertAidTM H Minus kits (Fermentas Life Science, Pittsburgh, PA, United States). One microliter of this cDNA was mixed with 2 × maxima SYBR Green PCR mix (12.5 μl) and RNase-free water (10.5 μl), and then 0.5 μl of each forward and reverse primer for the selected genes were added. The primers' sequences of genes coding TJ proteins, inflammatory (Toll-like receptors, TLR4), MUC-2, pro-inflammatory (interleukin-1β, IL-1β), and gut protective genes (3) are listed in Table 2. The amplification of real-time PCR was made with Rotor-Gene Q2 plex (Qiagen Inc., Valencia, CA, United States). Relative fold changes in the expression of target genes were calculated by the 2^−ΔΔCt^ method using glyceraldehyde-3-phosphatedehydrogenase (GAPDH) gene as an internal control gene to normalize target gene expression levels (24).

**Table 2:** Primer sequences and target genes used for real-time PCR reactions.

**Gene**	**Gene full name**	**Primer sequence (5^**′**^-3^**′**^)**
**Tight junction protein**		
Occludin		F-ACGGCAAAGCCAACATCTAC
		R-ATCCGCCACGTTCTTCAC
JAM-2	Junctional adhesion molecules	F-AGACAGGAACAGGCAGTGCT
		R-TCCAATCCCATTTGAGGCTA
**Gut protective**		
FABP-6	Fatty acids binding protein	GAGGACGCACCACGACTAAT TTTTCCCACCTTCCATTTTG
GLP-2	Glucagon-like peptide	F-CGTGCCACAGCCATTCTTA
		R-AGCGGCTCTGCAAATGATTA
**Inflammatory**		
TLR-4	Toll-like receptors	F-CTGCAGTTTCTGGATCTTTCAA
		R-TAAGCCATGGAAGGCTGCTA
IL-1β	Interleukin-1β	F-CAGCCCGTGGGCATCA
		R-CTTAGCTTGTAGGTGGCGATGTT
MUC-2	Mucin	F-ATTGAAGCCAGCAATGGTGT
		R-TTGTTGGCCTTGTCATCAAA
**House keeping**		
GAPDH	Glyceraldehyde-3-phosphate dehydrogenase	F-GGTGGTGCTAAGCGTGTTA R-CCCTCCACAATGCCAA
***C. jejuni*** **challenge**		
MDmapA1		Upper-CTATTTTATTTTTGAGTGCTTGTG
QCjmapAN		Lower-GCTTTATTTGCCATTTGTTTTATTA
		F-GGTTTTGAAGCAAAGATTAAAGG
		R-AAGCAATACCAGTGTCTAAAGTGC

### Challenge With *C. jejuni*

At the age of 35 days, 50 birds per group were orally infected with 10^8^ cfu of *C. jejuni* subsp. *jejuni* (ATCC 33291) per bird. *C. jejuni* count in feces at day 38 and day 42 and cecal contents at day 42 after slaughter of birds were detected by culture and RT-qPCR.

**Table 3:** Effects of dietary supplementation of *Glycyrrhiza* extract on growth performance of broiler chickens over 35 days.

**Parameters**	**Control**	**GE0.25**	**GE0.5**	**GE1**	**GE2**	***P*-value**	**SEM**
Initial body weight, g/bird	45	45	45	45	45	0.9	0.001
**Starter period**							
BW, g/bird	800^e^	833^c^	823^cd^	866^a^	837^bc^	<0.001	2.30
BWG, g/bird	755^e^	788^c^	778^cd^	821^a^	792^bc^	<0.001	3.03
FI, g/bird	1,208^a^	1,194^a^	1,134^b^	1,050^c^	1,072^d^	<0.001	63.27
FCR	1.60^a^	1.51^bc^	1.46^c^	1.28^d^	1.35c^d^	<0.001	0.06
**Grower–finisher period**							
BW, g/bird	2,164^e^	2,230^d^	2,378^ab^	2,681^a^	2,370^c^	<0.001	6.37
BWG, g/bird	1,364^e^	1,397^d^	1,555^ab^	1,815^a^	1,533^c^	<0.001	7.87
FI, g/bird	2,738^c^	2,538^e^	2,570^d^	2,852^a^	2,789^b^	<0.001	72.09
FCR	2.01^a^	1.82^b^	1.65^c^	1.57^d^	1.82^b^	<0.001	0.05
**All over period**							
BW, g/bird	2,164^d^	2,230^cd^	2,378^b^	2,681^a^	2,373^b^	<0.001	6.91
BWG, g/bird	2,119^d^	2,185^cd^	2,333^b^	2,636^a^	2,328^b^	<0.001	7.74
FI, g/bird	3,945^a^	3,731^c^	3,704^c^	3,902^ab^	3,861^b^	<0.001	152.80
FCR	1.86^a^	1.71^ab^	1.59^c^	1.48^d^	1.66^b^	<0.001	0.09
PER	2.57^e^	2.79^d^	3.01^b^	3.24^a^	2.89^c^	<0.001	0.04
Mortality, % (1–35)	2^a^	2^a^	1^b^	1^b^	1^b^	<0.001	0.03

*GE0.25 (basal diet supplemented with 0.25 g/kg Glycyrrhiza extract), GE0.5 (basal diet supplemented with 0.5 g/kg Glycyrrhiza extract), GE1 (basal diet supplemented with 1 g/kg Glycyrrhiza extract), GE2 (basal diet supplemented with 2 g/kg Glycyrrhiza extract). n = 10/replicate*.

BWG, body weight gain; FI, feed intake; FCR, feed conversion ratio.

a−e *Values within a row with different superscripts differ significantly at P < 0.05*.

Sampling post-infection: At 3 days post-infection (day 38): fresh fecal samples were collected immediately in a sterile tube for detection of *C. jejuni by* culture and RT-qPCR.

At 7 days post-infection (day 42): fecal and cecal samples (*n* = 20/group) were collected for detection of *C. jejuni by* culture and RT-qPCR and expression of genes coding for Toll-like receptors (TLR4), pro-inflammatory cytokines (interleukin-1β, IL-1β), and MUC-2.

#### Spread Plate Counting Method for *C. jejuni*

The number of viable *C. jejuni* in 1 g of fecal sample was confirmed and enumerated by plating duplicate dilutions on charcoal cefoperazone deoxycholate agar plates containing selective supplement (CCDA, SR155E, Oxoid) and incubated for 48 h at 42°C under microaerophilic conditions (25). The number of colonies was expressed as log^10^ CFU/g of sample.

One gram of cecal contents from each bird was collected after slaughtering and serially diluted for identification and quantification of *C. jejuni* as above.

#### Quantification of *C. jejuni* by Real-time qPCR

To ensure counting of viable and non-viable *C. jejuni*, RT-qPCR was tested (26).

DNA Extraction. Genomic DNA from chicken fecal and cecal samples were extracted by using a QIAamp DNA fast DNA stool kit (Qiagen, Hilden, Germany). The quality and quantity of DNA were detected using a Nano drop (Thermo-Fisher Scientific, MA, United States).

RT-qPCR. *C. jejuni* QCjmapAN primers (25) and MDmapA1 primers (27) as listed in Table 2 were used to quantify *C. jejuni* by RT-qPCR. To assemble the standard curve for *C. jejuni* quantification by RT-qPCR, we used the PCR product of the mapA gene using MDmapA1 primers. The copies of DNA molecules were calculated by making a standard curve using 10-fold dilutions of identified quantities of DNA plasmid (1 × 107 to 1 × 101 copies/μl). For RT-qPCR standard curves, functions showing the relationship between Ct and × (log^10^ DNA concentration in ng/μl) for the assays were used as described by Kurekci et al. (28). The quantitative PCR reaction for counting *C. jejuni* was done in a total volume of 10 μl containing 5 μl of the SYBR Green qPCR Master Mix (Qiagen, Australia), 2 μl of sample DNA, and 0.5 μl of the forward and reverse primers. The RT-qPCR results were analyzed using Rotor-Gene Q2 plex (Qiagen Inc., Valencia, CA, United States). The reaction condition for amplification of *C. jejuni* was 50°C for 2 min, 95°C for 2 min, and 40 cycles of 95°C for 15 s, 58°C for 30 s, and 72°C for 30 s, and a final extension of 72°C for 5 min.

The number of gene copy per gram of sample is equal to cell numbers as each *C. jejuni* cell was described to have a single copy from this gene (29).

### Statistics

The analysis of data was performed using the GLM procedure of SPSS, the experimental unit was a pen of broilers. The homogeneity among experimental groups was performed using Levene's test and normality using Shapiro–Wilk's test by using the model Y ik = μ + Li + eik, where Y ik = the observation, μ = the overall means, and Li = effect of groups such as G1 (group 1) (*j* = 1, 2, 3, 4, and 5) as 1 = control group, 2 = group fed 0.25 g/kg of GE, 3 = group fed 0.5 g/kg of GE, 4 = group fed 1 g/kg of GE, and 5 = group fed 2 g/kg of GE, and eik = random error. Tukey's test was used to test for significant differences between the mean values. Variation in the data was expressed as SEM and the significance was set at 0.05. There was no significant difference among pens within the same group. Data for each pen was calculated individually, with a total number of 10 pens within the group and a total 50 pens for all five groups in the experiment. Fecal and cecal cfu data were converted to log^10^ cfu numbers before analysis. All graphs were created using the GraphPad Prism software (Version 8, GraphPad Software Inc.). The fold change was measured by the following equation: (B–A)/A, where the lowest value is A and highest value is B.

## Results

### Growth Performance

The growth parameters during the experimental period are shown in Table 3. During the starter period, the most significant increase (*P* < 0.05) in body weight gain was observed in groups supplemented with 1 and 2 g/kg Glycyrrhiza extract (GE). Additionally, by increasing the levels of GE, the feed intake (FI) was significantly decreased (*P* < 0.05) during this period. The FCR was improved in all groups fed on different levels of dietary GE. During the finisher period, groups fed 0.5 and 1 g/kg of GE exhibited the highest body weight gain, while the highest feed intake was estimated in the group fed 1 g/kg of GE. Dietary inclusion of 0.5 and 1 g/kg of GE significantly improved the FCR. At 35 days of age, birds in groups fed 1 g/kg of GE showed a significant increase (*P* < 0.05) in final body weight gain (2636 g) followed by groups fed 0.5 and 2 g/kg of GE (2,333 and 2,328 g, respectively), as compared with groups fed 0.25 g/kg of GE and control diet (2,185 and 2,119 g, respectively). Birds fed 1 g/kg of GE had significantly better (*P* < 0.05) FCR and PER than the group fed 0.5 g/kg of GE when compared with other groups. Dietary supplementation of GE at different levels significantly decreased (*P* < 0.05) the total feed intake when compared with the control group. The mortality (days 1–35) was 2% for the control and 0.25 g/kg GE group and significantly decreased to 1% in groups fed 0.5, 1, and 2 g/kg of GE.

### TJ Protein Expression

**Figure 1:**
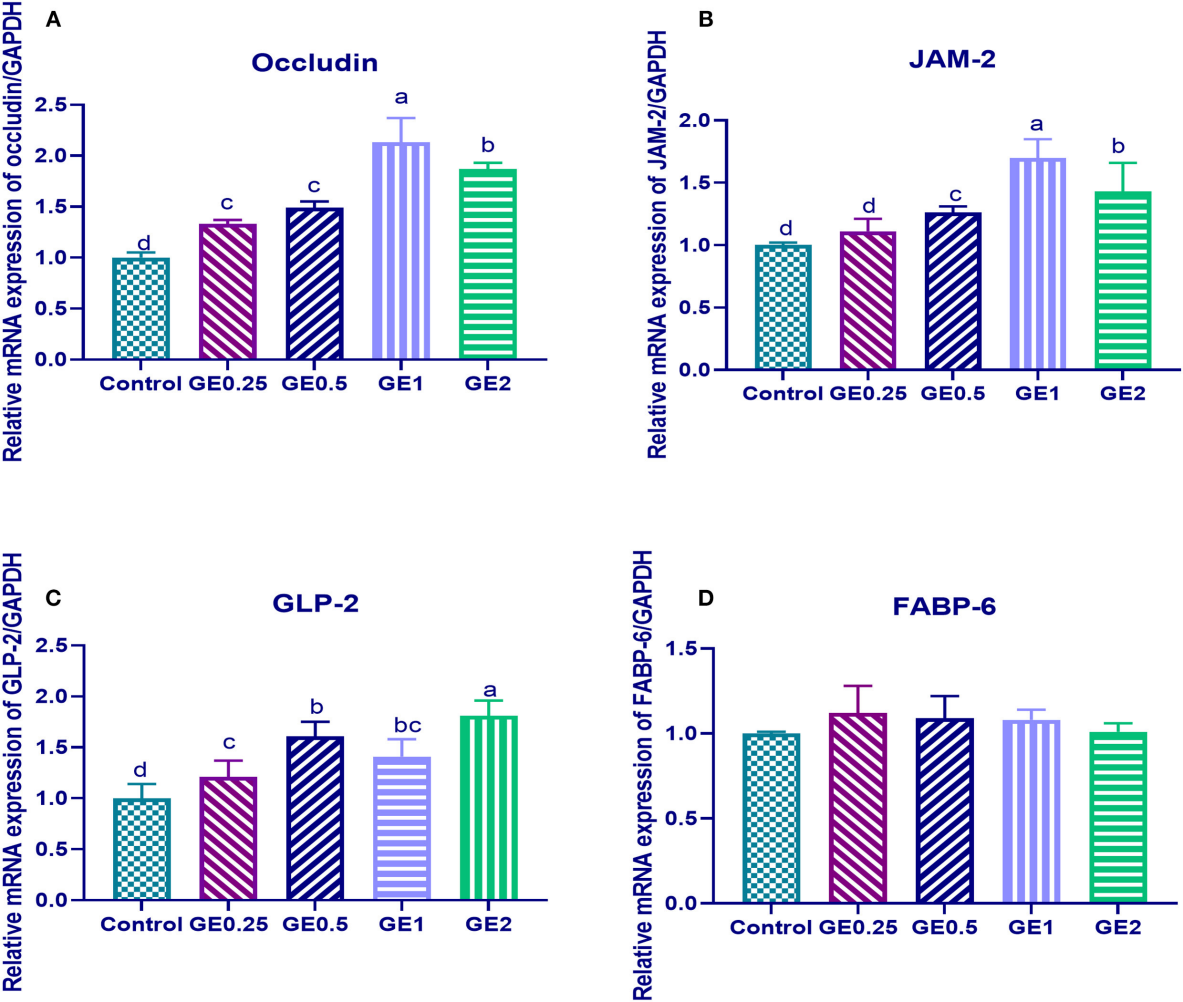
Effects of dietary supplementation of *Glycyrrhiza* extract on genes coding tight junction protein expression [occludin and junctional adhesion molecules (JAM-2)] and gut protective genes (GLP-2, FABP-6). Control referred to basal diet. GE0.25, GE0.5, GE1, and GE2 referred to basal diet supplemented with 0.25, 0.5, 1, and 2 g/kg *Glycyrrhiza* extract, respectively. Data represent mean values from 10 independent experiments; error bars indicate SE, *n* = 10. **(A)** Occludin expression was significantly upregulated in all groups supplemented with GE compared with the control group (*P* < 0.05). Occludin expression in GE0.25 and GE0.5 showing non-significant difference (*P* < 0.05). **(B)** JAM-2 expression was significantly upregulated in all groups supplemented with GE compared with the control group (*P* < 0.05) except for GE0.25 showing non-significant difference (*P* < 0.05). **(C)** GLP-2 expression was significantly upregulated in all groups supplemented with GE compared with the control group (*P* < 0.05). **(D)** FABP-6 expression in all groups supplemented with GE showing non-significant difference (*P* < 0.05) compared with the control group.

The expression of gene coding for occludin was significantly upregulated (*P* < 0.05) in all groups supplemented with GE when compared with the control group. Gene coding for JAM-2 was significantly upregulated (*P* < 0.05) in groups supplemented with 0.5, 1, and 2 g/kg of GE when compared with the control group. Moreover, upregulation of occludin and JAM-2 genes reached its peak with 0.2 and 0.3 fold changes in group fed 1 g/kg of GE and then their expression was downregulated in the group supplemented with 2 g/kg of GE (Figure 1).

### Gut Protective Gene Expression

Dietary supplementation of different levels of GE upregulated the expression of the GLP-2, gene and the highest expression was observed in the group fed 2 g/kg of GE with 0.8 fold change in comparison with the control group, while the expression of FABP was not significantly different among different groups (Figure 1).

### Gut Inflammatory Gene Expression and Numbers of *C. jejuni* in Fecal and Cecal Samples

#### Gut Inflammatory Gene Expression

**Figure 2:**
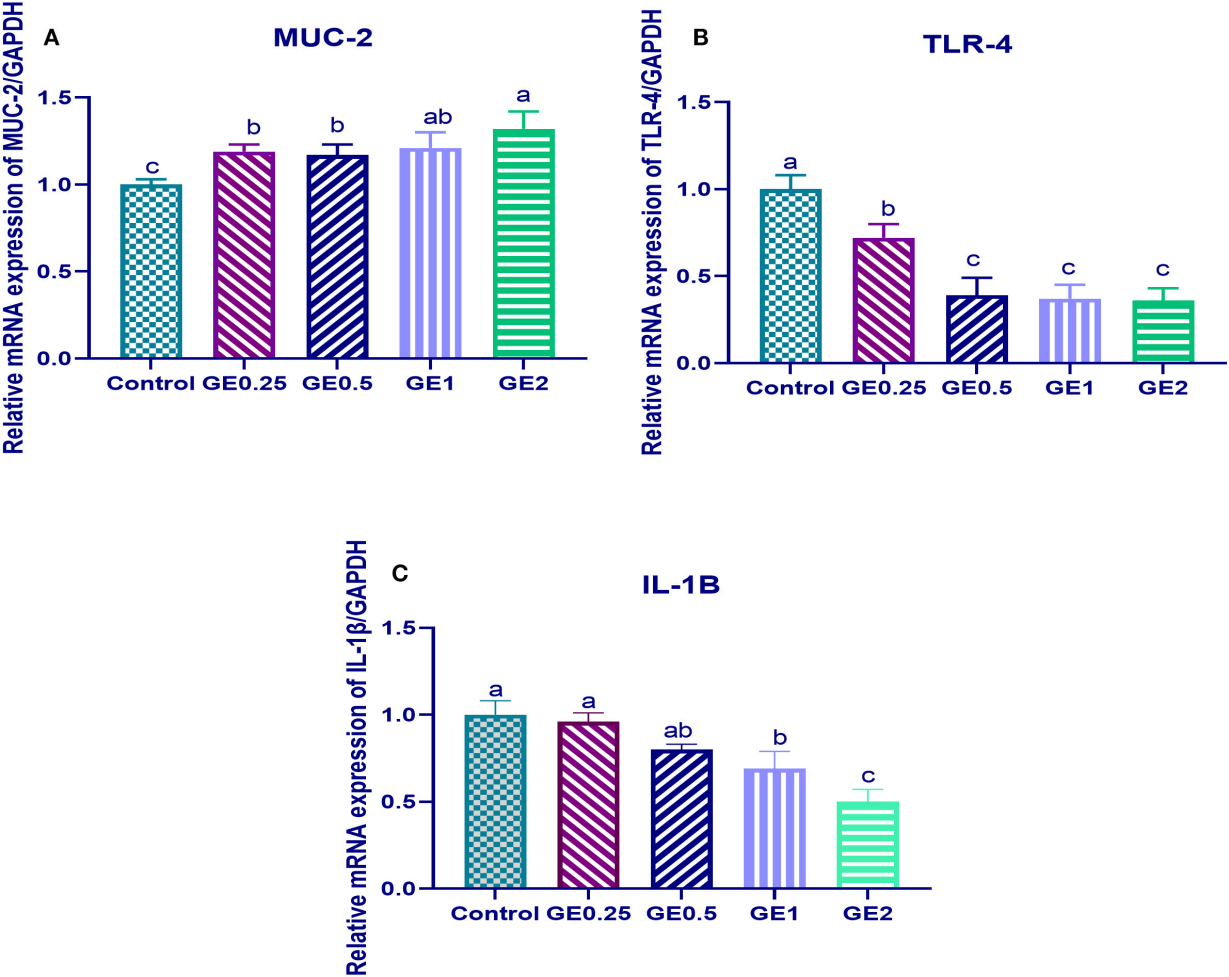
Effects of dietary supplementation of *Glycyrrhiza* extract on Mucin (MUC-2) and Toll-like receptors (TLR-4) 7 days post-infection. Control referred to basal diet. GE0.25, GE0.5, GE1, and GE2 referred to basal diet supplemented with 0.25, 0.5, 1, and 2 g/kg *Glycyrrhiza* extract, respectively. Data represent mean values from 10 independent experiments, error bars indicate SE, *n* = 50 per group. **(A)** MUC-2 expression was significantly upregulated in all groups supplemented with GE compared with the control group (*P* < 0.05). **(B)** TLR-4 expression was significantly downregulated in all groups supplemented with GE compared with the control group (*P* < 0.05). **(C)** IL-1β was significantly decreased in all groups supplemented with GE compared with the control group (*P* < 0.05) in a dose-dependent manner.

The expression of MUC-2 TLR-4 and IL-1β after challenge was presented in Figure 2. Gene expression of MUC-2 was significantly upregulated in groups supplemented with GE compared to the control group (*P* < 0.05). In contrast, TLR-4 was significantly downregulated after dietary GE supplementation (*P* < 0.05). Moreover, group supplemented with 2 g/kg of GE showed the most significant upregulation of MUC-2 (0.3 fold change) and downregulation of TLR-4 (1.5 fold change) when compared with the control group. The expression of the IL-1β gene was downregulated after dietary GE inclusion (*P* < 0.05) in a dose-dependent manner.

#### *C. jejuni* Quantification in Fecal and Cecal Samples

**Figure 3:**
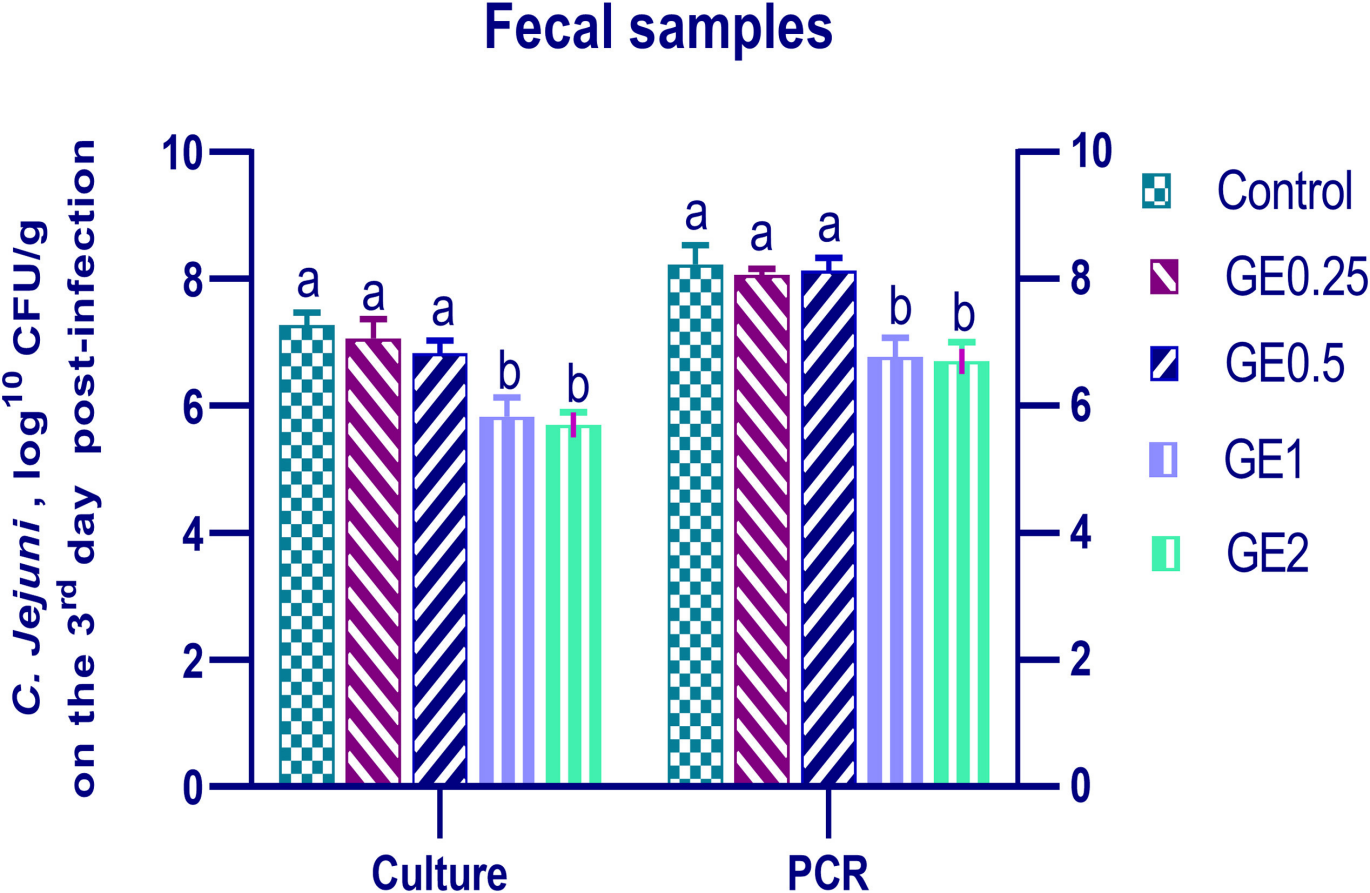
Effects of dietary supplementation of *Glycyrrhiza* extract on *C. jejuni* population in fecal samples 3 days post-infection. Control referred to basal diet. GE0.25, GE0.5, GE1, and GE2 referred to basal diet supplemented with 0.25, 0.5, 1, and 2 g/kg *Glycyrrhiza* extract, respectively. Data represent mean values from 10 independent experiments, error bars indicate SE, *n* = 20 per group. *C. jejuni* count was significantly decreased in all groups supplemented with GE compared with the control group (*P* < 0.05) except for GE0.25 and GE0.5 showing non-significant difference (*P* > 0.05).

**Figure 4:**
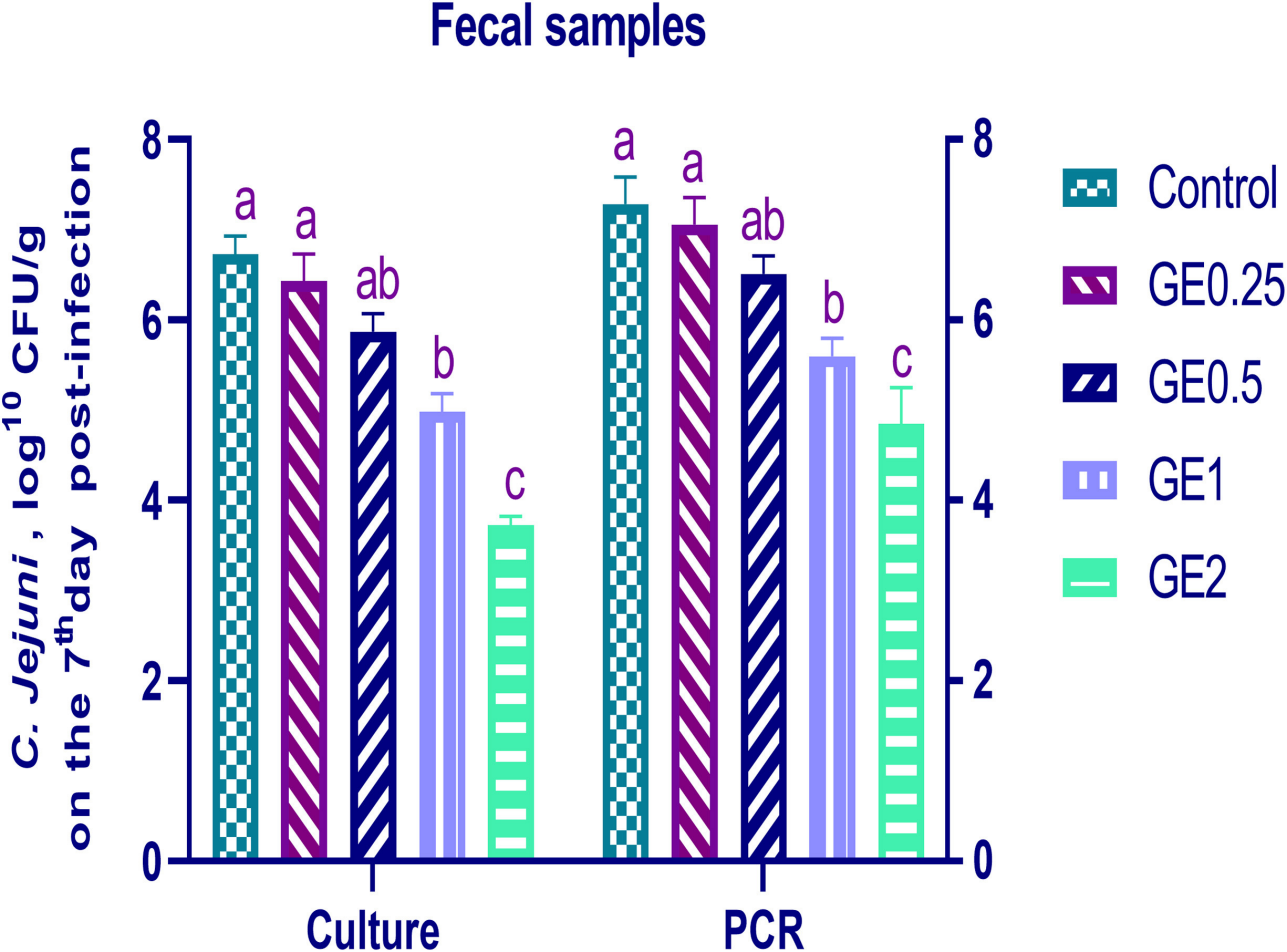
Effects of dietary supplementation of *Glycyrrhiza* extract on *C. jejuni* population in fecal samples 7 days post-infection (Culture and RT-qPCR). Control referred to basal diet. GE0.25, GE0.5, GE1, and GE2 referred to basal diet supplemented with 0.25, 0.5, 1, and 2 g/kg *Glycyrrhiza* extract, respectively. Data represent mean values from 10 independent experiments, error bars indicate SE, *n* = 20 per group. *C. jejuni* count was significantly decreased in groups supplemented with 1 and 2 g/kg GE compared with the control group (*P* < 0.05).

**Figure 5:**
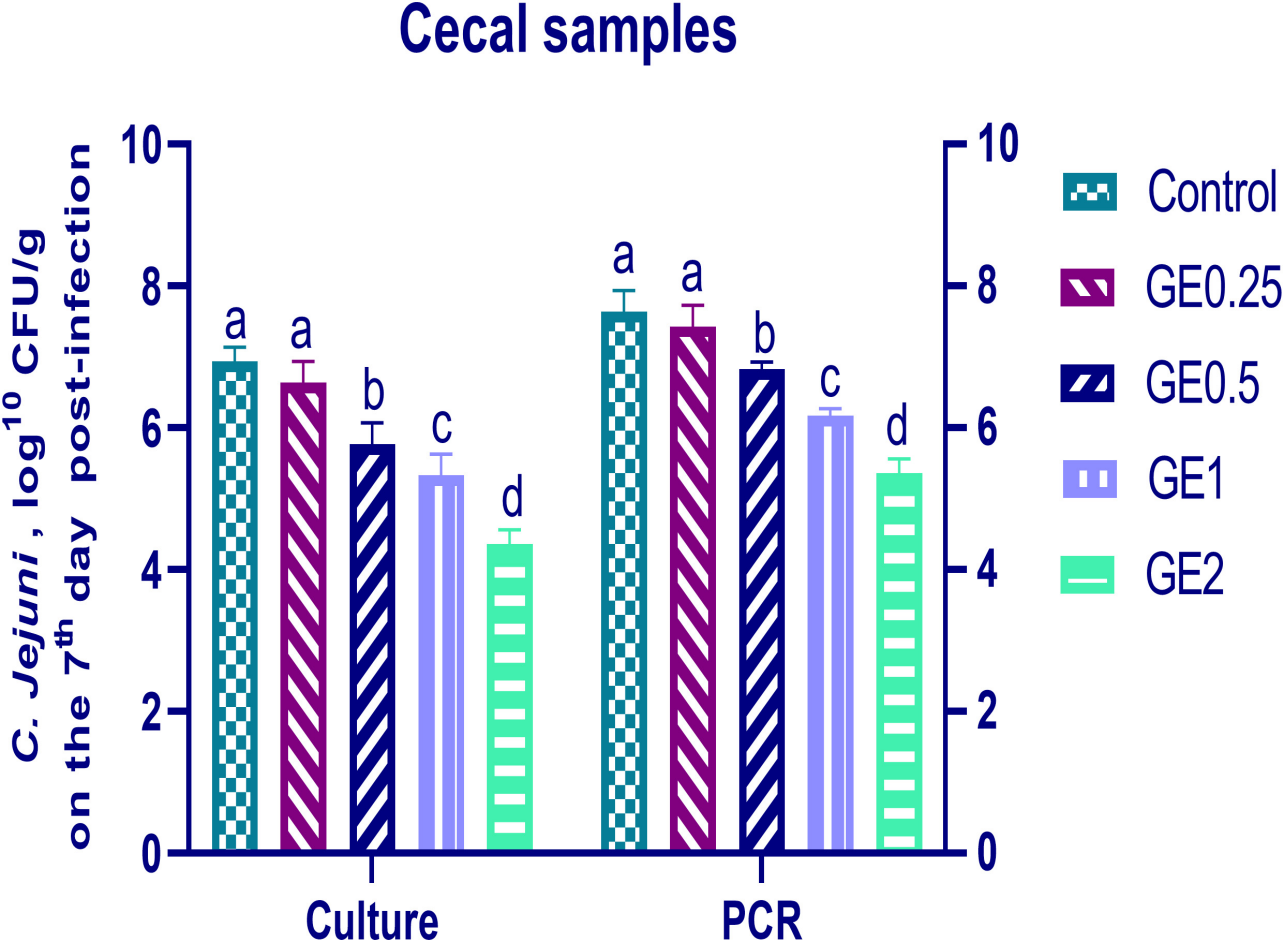
Effects of dietary supplementation of *Glycyrrhiza* extract on *C. jejuni* population in cecal samples 7 days post-infection (Culture and RT-qPCR). Control referred to basal diet. GE0.25, GE0.5, GE1, and GE2 referred to basal diet supplemented with 0.25, 0.5, 1, and 2 g/kg *Glycyrrhiza* extract, respectively. Data represent mean values from 10 independent experiments, error bars indicate SE, *n* = per 20. *C. jejuni* count was significantly decreased in all groups supplemented with GE compared with the control group (*P* < 0.05) except for GE0.25 showing non-significant difference (*P* < 0.05).

The collected fecal samples at day 35 before challenge with *C. jejuni* were free from *C. jejuni* [the count was less than the minimum detection limit (1 × 10^4^ cfu/g)]. *C. jejuni* count 3 days post-infection by traditional culture and RT-qPCR analysis was significantly decreased (*P* < 0.05) in groups supplemented with 1 and 2 g/kg of GE, while inclusion of GE (0.25 and 0.5 g/kg) showed a lower tendency to decrease the number of *C. jejuni* with non-significant difference compared with the control group (Figure 3). The count of *C. jejuni* in fecal samples 7 days post-infection either by culture or by RT-qPCR was significantly (*P* < 0.05) decreased by increasing levels of GE in the diet of broiler chickens. In addition, the lowest *C. jejuni* count in traditional culture and RT-qPCR analysis was observed in the group fed 2 g/kg of GE (3.72 and 4.85 log^10^ CFU/g, respectively) (Figure 4). The loads of *C. jejuni* in ceca of chicken 7 days post-infection either by culture or by RT-qPCR were significantly (*P* < 0.05) influenced by dietary inclusion of GE as the birds that received higher levels of GE (2 g/kg) had less *C. jejuni* count (4.36 and 5.36 log^10^ CFU/g, respectively) compared with the control group (Figure 5).

## Discussion

There is a growing interest in studying the beneficial effects of functional plant extracts for controlling diseases and improving broiler performance. *G. glabra* (licorice, GE) is one of the most used herbal medicinal plants, and its extract is currently used in food and pharmaceutical industries as well as in the manufacture of food supplements and functional foods (30). Additionally, modern pharmacological studies extensively described that flavonoids such as glabridin, liquiritin apioside, liquiritigenin, isoliquiritoside, isoliquiritigenin, and triterpene saponins such as glycyrrhizic acid were the main bioactive compounds supporting the biological effects of *G. glabra*. In the current study, the use of *G. glabra* extract powder at 0.25, 0.5, and 1 g/kg feed has improved the growth performance of broiler chickens over 35 days. Previous studies reported that dietary GE enhanced the body gain and feed efficiency in broiler chickens (31) and it has been recommended to be an alternative to in-feed antibiotics growth promoters in broiler chickens (32, 33). GE has a dual role in promoting growth of broiler chickens: one was to improve digestion (34) and the other may be related to its active components, mainly flavonoids, pentacyclic triterpene, and glycyrrhizin (35).

In agreement, the use of triterpenoids as glycyrrhizic acid at the 60 g/ml level in water decreased mortality rate, increased body weight gain, and improved FCR of broilers chickens in comparison with the control group (36).

Additionally, dietary supplementation of flavonoid-rich plant extracts has been reported to improve the growth performance of broiler chickens (37–39). This improved growth performance is likely due to the beneficial effect of flavonoids on gut functions (40).

The beneficial effect of *G. glabra* flavonoids such as glabridin, liquiritin apioside, liquiritigenin, and isoliquiritoside on broilers' growth performance may result from upregulation of the growth hormone and the hepatic growth hormone receptor, which increases the concentration of insulin-like growth factor 1, thus promoting animal growth (41).

The cells of the intestinal barrier are connected by TJ complexes consisting of proteins including occludin and junctional adhesion molecules (42). Several dietary factors displayed protective effects on the intestinal barrier (9). Maintaining the integrity of the epithelial cell barrier and nutrient utilization by GE in broilers are not fully understood until now. In our study, the upregulation of occludin and JAM-2 gene expression after dietary inclusion of GE indicated the beneficial effects of GE on gut barrier function. In accordance, the upregulation of occludin gene expression was accounted for by improving the functions of TJ barrier and preventing its damage (43). In addition, GE enhanced the recovery of intestinal barrier function in porcine cell culture through upregulating the expression of TJ proteins claudin-4 and occludin (42). The mode of action of *Glycyrrhiza* extract may be due to its content from flavonoids such as liquiritin apioside, liquiritigenin, isoliquiritoside, isoliquiritigenin, and liquiritin (44). Previous researchers suggested that flavonoids may improve intestinal barrier functions through the following mechanisms: (1) maintaining the structure of the intestinal epithelial barrier (45), (2) inhibiting inflammatory signaling such as NF-κB and extracellular pathway-regulated kinases, (3) decreasing oxidative stress by nicotinamide adenine dinucleotide phosphate oxidase expression, and (4) upregulating gut hormones such as glucagon-like peptide (GLP-2) that improves intestinal barrier function (46).

The gut epithelium has about 90% of the absorptive epithelial cells that express high levels of enteroendocrine cells responsible for gut peptide secretion such as GLP-1 and GLP-2 (47). Also, Brubaker (48) stated that GLP-2 is produced in the intestine and exerts multiple effects on the GIT to adjust food intake and to control digestion and absorption by signaling feedback to the brain. In addition, this peptide has been shown to decrease intestinal inflammation (49), permeability (50), and bacterial translocation (51). On the other hand, FABP is mainly expressed in the intestinal epithelium, which is thought to accelerate long-chain fatty acid uptake from the digesta into enterocytes (52) and protect against the cytotoxic effects of free cellular fatty acids; thus, it has an important role in enterocyte protection (3). In contrast the overexpression of FABP indicated intestinal epithelial cell damage (53). The modulatory effect of GE on gut protective genes (upregulation of GLP-2 and downregulation of FABP) may be attributed to its flavonoid content with an antioxidant effect by activating the expression of GLP-2 and protecting intestinal epithelium from inflammation (54). In addition, flavonoids reduced the expression of FABP and thus protect the intestinal epithelial cells from damage caused by increased free cellular fatty acids (55).

Previous studies have described that inflammatory cytokines and bacterial antigens can affect TJ protein expression, thus altering TJ functions (56). Pattern recognition receptors (PRRs) can initiate immune response by recognizing microbial antigens known as pathogen-associated molecular patterns (57). These PRRs comprise Toll-like receptors (TLRs) placed on the cell membrane and nucleotide binding oligomerization domain (NOD)-like receptors (NLRs) present in the cytoplasm (58). Activation of TLR4 by bacterial lipopolysaccharides can modulate inflammatory reactions via myeloid differentiation factor 88 (MyD88)-independent signaling cascades, and MyD88-dependent signaling eventually causes production of pro-inflammatory cytokines (59, 60). The results after challenge described that the use of *Glycyrrhiza* extract in broiler diet has been shown to downregulate expression of Toll-like receptors (TLR-4) in a dose-dependent manner. In accordance, the protection of intestinal epithelium from inflammation was evidenced by decreasing expression levels of TLR-4 that played an important role in stimulation of intracellular signals and subsequently modified transcriptional expression of several inflammatory mediators (61). Additionally, pro-inflammatory cytokines could induce TJ disruption, resulting in increased intestinal permeability (62). The greater reduction of TLR4 and pro-inflammatory cytokines (IL-1β) by GE supplementation even after *C. jejuni* infection indicated its anti-inflammatory effect. In agreement with this observation, triterpene and flavone (glabridin, licochalcone, and liquiritigenin) contents of GE can downregulate inflammatory mediators that play a key role in the development of diseases (63) and consequently reduce intestinal inflammation.

Thin or defective intestinal mucus layer might result in a higher GI permeability and an increase in bacterial adhesion to the mucosal epithelium causing intestinal pathological alterations (64). Lipopolysaccharide found in gram-negative bacterial cell walls such as *C. jejuni* can prompt GI inflammation via pro-inflammatory cytokine activation and secretion of inflammatory mediators (65), which, in turn, may reduce the expression of junction proteins (e.g., claudins, JAM-2, occludin, and ZO1) and intestinal MUC-2 (66). The results after *C. jejuni* infection described that increasing GE level was accompanied by increasing expression of the MUC-2 gene. Many phytogenics may enhance gene expression responsible for mucin production in goblet cells (15) and protect the gastrointestinal mucus layer (67). MUC-2, together with water and small amounts of related mucin proteins, polymerizes into a gel that provides an unstirred insoluble mucous barrier helping to defend the GI epithelium by limiting large molecules from directly attaching to the intestinal epithelium, including bacteria (68). In the same line, licorice-derived compounds may increase prostaglandin concentration in the GIT, which stimulates the mucus secretion from the stomach and consequently initiates healing effects (69). Disruption in the integrity of GI barrier allows entrance of luminal antigenic elements that activate immune response and inflammation (63).

The effective role of GE against *S. aureus* (70), *E. coli* (71), and *Pseudomonas aeruginosa* (72) was investigated. However, the activity of GE against *C jejuni* infection was not fully understood in broilers. In our study, decreasing *C jejuni* colonization was observed following supplementation of higher levels of GE. The antimicrobial activity of GE can be attributed to the content of triterpene and three flavones (glabridin, licochalcone, and liquiritigenin). These agents can reduce microbial gene expression, prevent microbial growth, and reduce toxin secretion from the microbe (73). On the other hand, these previous compounds downregulated inflammatory mediators that played a key role in the development of diseases (63). Additionally, licorice root extracts have been examined in the gastritis treatment induced by *Helicobacter pylori* and proved promising results (74). Similarly, an *in vitro* study described that *G. glabra* had the highest antiadhesion activity against *C. jejuni* (20). Upregulating the genes encoding for TJ protein before infection and MUC-2 (Figure 2) after infection indicated the protective effects of GE against *C. jejuni* colonization.

## Conclusion

Using *Glycyrrhiza* extract increased broiler performance by affecting molecular mechanisms responsible for intestinal integrity and protection of enterocytes from inflammation. Another benefit from using GE was decreasing *C. jejuni* shedding after challenge, which can reduce the incidence of infection for humans in broiler slaughterhouses. Overall, the present work demonstrated that GE inclusion in poultry diets at the 1 g/kg level can be used as a growth promoter and therapeutic tool against *C. jejuni* infection.

## Data Availability Statement

The raw data supporting the conclusions of this article will be made available by the authors, without undue reservation.

## Ethics Statement

The animal study was reviewed and approved by Institutional Animal Care and Use committee of Faculty of Veterinary Medicine at Zagazig University.

## Author Contributions

All authors shared in designing the study, methodology, data collection and analysis, statistical analysis, and writing the manuscript.

## Conflict of Interest

The authors declare that the research was conducted in the absence of any commercial or financial relationships that could be construed as a potential conflict of interest.

## Abbreviations

AOAC: Association of Official Agricultural Chemists
GE: Glycyrrhiza extract
GIT: Gastrointestinal tract
GLP-2: Glucagon-like peptide
FABP-6: Fatty acid-binding protein
FCR: Feed conversion ratio
IGF-1: Insulin like growth factor-1
IL-1β: Interleukin 1, beta
JAM: Junctional adhesion molecules
MUC-2: Mucin-2
PER: Protein efficiency ratio
PFA: Phytogenic feed additives
PBS: Phosphate buffer saline
TJ: Tight junction
TLR-4: Toll-like receptors.
